# Supplementary material for: Gender and Professional Role Differences in Chilean Educational Personnel’s Perceptions of School Climate and Well-Being
Source: Behav Sci (Basel). 2025 Oct 24;15(11):1447. doi: 10.3390/bs15111447 (PMC12649673; doi:10.3390/bs15111447)
Supplement: Supplementary file 1 [file behavsci-15-01447-s001.zip › behavsci-3797204-supplementary.pdf]

behavioral sciences

MDPI

Supplementary Material

Gender and Professional Role Differences in Chilean Educational Personnel's Perceptions of School Climate and Well-Being

Flavio Muñoz-Troncoso [1,2,3], Enrique Riquelme-Mella [1,*], Ignacio Montero [4] and Gerardo Muñoz-Troncoso [5,*]

[1] Faculty of Education, Universidad Católica de Temuco, Temuco 4810296, Chile
[2] International Observatory on School Climate and Violence Prevention (IOSCVP), 41004 Sevilla, Spai
[3] Faculty of Social Sciences and Arts, Universidad Mayor, Temuco 4801043, Chile
[4] Faculty of Psychology, Universidad Autónoma de Madrid, 28049 Madrid, Spain
[5] Faculty of Philosophy and Humanities, Universidad Austral de Chile, Valdivia 5110566, Chile
* Correspondence: eriquelme@uct.cl (E.R.-M.); gerardo.munoz01@uach.cl (G.M.-T.)

Table S1. Distribution of Participants by Age Groups.

Age groups	Frequency	Percent Valid	Percent	Cumulative Percent
18–25 years	338	4	4	4
26–30 years	1,551	18.2	18.2	22.1
31–35 years	1,666	19.5	19.5	41.6
36–40 years	1,429	16.7	16.7	58.4
41–45 years	889	10.4	10.4	68.8
46–50 years	668	7.8	7.8	76.6
51–55 years	812	9.5	9.5	86.1
56–60 years	748	8.8	8.8	94.9
61 years and older	435	5.1	5.1	100
Total	8,536	100	100	

Source: Prepared by the authors.

Table S2. Distribution of Participants by Macrozone.

Macrozone	Frequency	Percent Valid	Percent	Cumulative Percent
North	589	6.9	6.9	6.9
Central	2293	26.9	26.9	33.8
South–Central	2931	34.3	34.3	68.1
South	696	8.2	8.2	76.3
Far South	108	1.3	1.3	77.5
Metropolitan	1919	22.5	22.5	100
Total	8,536	100	100	

Source: Prepared by the authors.

Table S3. Distribution of Participants by Region.

Region	Frequency	Percent Valid	Percent	Cumulative Percent
Arica y Parinacota	43	0.5	0.5	0.5
Tarapacá	90	1.1	1.1	1.6
Antofagasta	346	4.1	4.1	5.7
Atacama	110	1.3	1.3	7.0
Coquimbo	394	4.6	4.6	11.6
Valparaíso	1899	22.2	22.2	33.8
Metropolitana	1919	22.5	22.5	56.3
O'Higgins	548	6.4	6.4	62.7
Ñuble	567	6.6	6.6	69.3
Maule	754	8.8	8.8	78.1
Biobío	1062	12.4	12.4	90.5
La Araucanía	534	6.3	6.3	96.8
Los Ríos	24	0.3	0.3	97.1
Los Lagos	138	1.6	1.6	98.7
Aysén	7	0.1	0.1	98.8
Magallanes	101	1.2	1.2	100.0
Total	8,536	100	100	

Source: Prepared by the authors.

Table S4. Distribution of Participants by Municipality.

Municipality	Frequency	Percent Valid	Percent	Cumulative Percent
Aisén	7	0.1	0.1	0.1
Alto Hospicio	90	1.1	1.1	1.2
Antofagasta	294	3.4	3.4	4.6
Arica	43	0.5	0.5	5.1
Buin	166	1.9	1.9	7.0
Cabildo	87	1	1	8.0
Calera	276	3.2	3.2	11.2
Carahue	28	0.3	0.3	11.5
Cauquenes	63	0.7	0.7	12.2
Chañaral	40	0.5	0.5	12.7
Chillán	236	2.8	2.8	15.5
Chillán Viejo	58	0.7	0.7	16.2
Coihueco	100	1.2	1.2	17.4
Colbún	21	0.2	0.2	17.6
Colina	62	0.7	0.7	18.3
Concepción	60	0.7	0.7	19.0
Copiapó	70	0.8	0.8	19.8
Coquimbo	80	0.9	0.9	20.7

Coronel	25	0.3	0.3	21.0
Curicó	155	1.8	1.8	22.8
El Monte	26	0.3	0.3	23.1
Freire	15	0.2	0.2	23.3
Gorbea	25	0.3	0.3	23.6
Graneros	58	0.7	0.7	24.3
Hijuelas	78	0.9	0.9	25.2
Hualpén	90	1.1	1.1	26.3
Huechuraba	142	1.7	1.7	28.0
Isla de Maipo	37	0.4	0.4	28.4
La Florida	66	0.8	0.8	29.2
La Ligua	47	0.6	0.6	29.8
La Pintana	179	2.1	2.1	31.9
La Serena	153	1.8	1.8	33.7
La Unión	24	0.3	0.3	34.0
Lampa	43	0.5	0.5	34.5
Limache	43	0.5	0.5	35.0
Lo Espejo	186	2.2	2.2	37.2
Longaví	40	0.5	0.5	37.7
Los Ángeles	278	3.3	3.3	41.0
Lota	272	3.2	3.2	44.2
Macul	24	0.3	0.3	44.5
Melipilla	42	0.5	0.5	45.0
Molina	74	0.9	0.9	45.9
Mostazal	72	0.8	0.8	46.7
Mulchén	28	0.3	0.3	47.0
Nueva Imperial	43	0.5	0.5	47.5
Ñuñoa	172	2	2	49.5
Olmué	49	0.6	0.6	50.1
Osorno	43	0.5	0.5	50.6
Ovalle	161	1.9	1.9	52.5
Parral	72	0.8	0.8	53.3
Pedro Aguirre Cerda	72	0.8	0.8	54.1
Peñaflor	42	0.5	0.5	54.6
Peñalolén	155	1.8	1.8	56.4
Pichidegua	115	1.3	1.3	57.7
Pudahuel	26	0.3	0.3	58.0
Puente Alto	88	1	1	59.0
Puerto Montt	53	0.6	0.6	59.6
Puerto Saavedra	72	0.8	0.8	60.4
Punta Arenas	101	1.2	1.2	61.6
Purranque	42	0.5	0.5	62.1
Putaendo	21	0.2	0.2	62.3
Quilicura	134	1.6	1.6	63.9

Quillota	102	1.2	1.2	65.1
Quilpué	197	2.3	2.3	67.4
Quinta de Tilcoco	24	0.3	0.3	67.7
Quinta Normal	73	0.9	0.9	68.6
Quintero	53	0.6	0.6	69.2
Rancagua	110	1.3	1.3	70.5
Recoleta	27	0.3	0.3	70.8
Retiro	85	1	1	71.8
Sagrada Familia	63	0.7	0.7	72.5
San Antonio	451	5.3	5.3	77.8
San Carlos	173	2	2	79.8
San Clemente	22	0.3	0.3	80.1
San Felipe	354	4.1	4.1	84.2
San Fernando	39	0.5	0.5	84.7
San Pedro	29	0.3	0.3	85.0
San Pedro De La Paz	173	2	2	87.0
San Ramón	48	0.6	0.6	87.6
San Vicente	94	1.1	1.1	88.7
Santa Cruz	36	0.4	0.4	89.1
Talagante	80	0.9	0.9	90.0
Talca	85	1	1	91.0
Talcahuano	136	1.6	1.6	92.6
Temuco	97	1.1	1.1	93.7
Tocopilla	52	0.6	0.6	94.3
Valparaíso	141	1.7	1.7	96.0
Victoria	86	1	1	97.0
Villarrica	168	2	2	99.0
Yerbas Buenas	74	0.9	0.9	100.0
Total	8,536	100	100	

Note: Municipalities are presented in alphabetical order.

Source: Prepared by the authors.
